# Role of Wood Fibers in Tuning Dynamic Rheology, Non-Isothermal Crystallization, and Microcellular Structure of Polypropylene Foams

**DOI:** 10.3390/ma12010106

**Published:** 2018-12-30

**Authors:** Yongming Song, Youyong Wang, Hao Li, Qiling Zong, Ailing Xu

**Affiliations:** Key Laboratory of Bio-based Material Science and Technology (Ministry of Education), Material Science and Engineering College, Northeast Forestry University, Harbin 150040, China; wangyouyong2018@163.com (Y.W.); leehao1127@163.com (H.L.); zongqiling1239@163.com (Q.Z.); xuailingxal@163.com (A.X.)

**Keywords:** polypropylene, wood fiber, rheology, non-isothermal crystallization, supercritical CO_2_, microcellular foaming

## Abstract

Microcellular polypropylene (PP)/wood fiber composite foams were fabricated via batch foaming assisted by supercritical CO_2_ (scCO_2_). Effects of wood fibers on rheology, crystallization, and foaming behaviors of PP were comprehensively investigated. The obtained results showed that the incorporation of wood fibers increased the complex viscosity and the storage modulus of the PP matrix. Jeziorny’s model for non-isothermal crystallization kinetics indicated that wood fibers did not change the crystal growth. However, the crystallization rate of the PP matrix was decreased to a certain extent with increasing wood fiber loadings. The wood fiber exerts a noticeable role in improving the cell density and reducing the cell size, despite decreasing the expansion ratio. Interestingly, a “small-sized cells to large-sized cells” gradient cell structure was found around the wood fibers, implying cell nucleation was induced at the interface between wood fiber and PP matrix. When wood fiber loadings were specifically increased, a desirable microcellular structure was obtained. However, further increasing the wood fiber loadings deteriorated the cell structure. Moreover, the crystallinity of the composite foams initially decreased and then slightly increased with increasing wood fiber loadings, while the crystal size decreased.

## 1. Introduction

Wood fiber reinforced plastic composites (WPCs) have experienced rapid growth and have become a viable alternative to replace pure wood and plastic products. This can be attributed to their low cost, preferable appearance and performance, and environmental friendliness [1,2,3,4,5]. Based on these superior properties, WPCs have been utilized in a wide range of applications, including indoor and outdoor construction materials, furnishings, and in the automobile industry [6,7]. However, there are several drawbacks of WPCs that constrict their applications in many areas, such as their low impact resistance, and high density compared to the unfilled thermoplastics and natural wood. In recent years, microcellular foaming processing has been proposed as a suitable strategy that could improve the toughness while reducing the material density by producing a microcellular structure in the WPCs [8,9,10]. 

Microcellular foaming processing uses chemical or physical blowing agents to produce a porous structure, with a cell size below 10 μm and a cell density above 10^9^ cells/cm^3^ [11,12,13]. These particular characteristics equip microcellular foams with a significant increase in thermal stability, toughness, and elongation at break, in comparison to traditional foams [13,14]. Supercritical fluids possess the same solubility as liquids, and their diffusion coefficient is similar to that of gases [15], which could be easily dissolved and diffused into the thermoplastic matrix. Among these, supercritical carbon dioxide (scCO_2_) has been considered as an ideal physical blowing agent for microcellular foaming, since it is inexpensive, non-toxic, and leaves no chemical residue [16,17]. Moreover, scCO_2_ has a higher solubility in the polymer matrix than other physical foaming agents (e.g., supercritical N_2_ or He). Therefore, using scCO_2_ as foaming agent to produce the microcellular structure in the WPCs is a promising approach.

During the microcellular foaming process, melt viscosity and crystallization behaviors are vitally important parameters, which affect the solubility of blowing agents, cell nucleation, cell growth, and cell stabilization [18,19]. During formulation, wood fibers influence the polymer foaming processing because wood fibers can change the melt rheology, crystallization, and the corresponding foaming behaviors, which significantly affect the microcellular structure of polymer/wood fiber composite foams [19,20,21]. Addition of wood fibers increases the viscosity of the polymer matrix, and the high melt viscosity resists cell growth, resulting in smaller cell size and lower expansion ratio to some extent. Previous research verified that wood fibers could act as heterogeneous nucleation sites and promote the overall cell nucleation density, which in turn improves cell density [20,22,23]. In addition, the wood fibers could act as reinforcement fillers, leading to improved specific stiffness and strength of the composite foams [24,25]. Simultaneously, the wood fibers could improve the cell structure including smaller cell size, higher foam density, and thicker cell walls. Rodrigue et al. [26] reported that the addition of 5 wt.% wood flour resulted in improvement of cell nucleation and reduction of cell size during the chemical extrusion foaming, the cell diameter was 76 μm, and the cell density was 8 × 10^6^ cells/cm^3^. However, when exposed to heat, wood fibers release water vapor and volatile components that could negatively affect the development of the cell structure in foamed composites [20]. Faruk et al. [27] prepared polypropylene (PP)/wood fiber composite foams with high wood fiber loadings through foam injection molding (FIM) and extrusion foaming processing, respectively. The obtained composite foams had a poor cell structure and the FIM process showed better achievement compared to the extrusion process in maximum properties. The previous research reported that high wood flour loadings reduced the solubility of scCO_2_, destroyed the continuity of the polymer matrix, and accelerated gas escape during foaming, all of which were not conducive to the formation of the desirable microcellular structure. The relationships between microcellular morphology, processing conditions and mechanical properties of foamed WPCs have been studied [6,14]. However, relatively few studies have discussed the relationship between wood fiber induced rheology behaviors and the final obtained cell structure. In addition, it remains challenging to introduce a desirable microcellular structure in the WPCs, especially in the high wood fiber loadings, due to the low melt strength and the deteriorated continuous phase structure of the polymer matrix [28]. In this study, microcellular PP/wood fiber composite foams were fabricated with high wood fiber loadings via the batch foaming process based on a high melt strength PP matrix. The relationship between wood fiber tailored dynamic rheology as well as the non-isothermal crystallization behaviors of PP and the foam structure was comprehensively investigated. Moreover, the cell distribution characteristics around the wood fibers and the heterogeneous nucleation role of the wood fibers were investigated and first illustrated by the theory of the segment-dynamics heterogeneity. 

## 2. Materials and Methods

### 2.1. Materials

The polymer matrix used in this study was high melt strength PP, supplied by LyondellBasell (PF814, Lyondell, Holland) with a melt flow index of 2.5 g/10min (ASTM D1238) [29] and a density of 0.9 g/cm^3^. Poplar (*populus*) wood fibers (WF,) with a particle size about 145–210 μm were supplied by Sanqi Wood Fiber Agency (Linyi, China). Industrial grade talc was used as the cell nucleating agent and was provided by Shanghai Smart Chemical Reagent Co., Ltd. (Shanghai, China), with an average size of 4.2 μm. Commercial grade maleic anhydride-grafted polypropylene (PP-g-MA) was used as compatibilizer and was purchased from Shanghai Risheng New Technology Development Co., Ltd. (Shanghai, China). Polyethylene wax (PE wax) was used to enhance the dispersion of wood fibers and was purchased from Shandong Qilu Petrochemical Engineering Co., Ltd. (Zibo, China). Commercial grade carbon dioxide (purity 99.9%) was used as physical blowing agent and was purchased from Shanghai Gas Co., Ltd. (Shanghai, China).

### 2.2. Preparation of PP/Wood Fiber Composites

Firstly, the wood fibers were oven-dried at 103 ± 5 °C for 12 h to achieve a moisture level below 2%. Secondly, PP (100, 90, 80, 70, and 60 wt.%), well-dried wood fiber (0, 10, 20, 30, and 40 wt.%), Talc (4 wt.%) based on PP matrix, PP-g-MA (5 wt.%) based on wood fiber, and PE wax (2 wt.%) based on wood fiber were put into a high-speed blender and were mixed for 5 min. The choice of talc, compatibilizer, and PE wax fraction was based on our previous research [2,23]. Then, a co-rotating twin-screw extruder was used to further mix the melt at a rotation speed of about 50 rpm. The extrudates were compressed into sheets at 180 °C and at 12 MPa for 4 min, to about 2 mm thickness. After 1 to 2 h, the compression-molded sheets were cut into 10 mm × 20 mm rectangular specimens in preparation for the batch foaming.

### 2.3. Fabrication of Polypropylene/Wood Fiber Composite Foams

Microcellular foaming was carried out in a high-pressure autoclave. The initial weights of the composite sheets were measured with a digital balance scale at an accuracy of 0.0001 g. Then, the samples were saturated with scCO_2_ at 16 MPa and 160 °C for about 30 min. To obtain microcellular composite foams, the high-pressure vessel was instantaneously depressurized to atmospheric pressure at a rate of about 40 MPa/s. The foamed specimens were taken out of the high-pressure vessel and cooled to the temperature of ambient air to allow for cell solidification and shaping.

### 2.4. Rheology Characterization

To characterize the rheology behavior of PP and the PP/wood fiber composites, a rotational rheometer AR2000ex (TA Instruments, New Castle, PA, USA) with a 25 mm parallel disk and a 2 mm gap was used. At first, the dynamic strain sweep test was conducted to identify the strain limit of the linear viscoelastic region. Afterwards, the dynamic strain sweep test was carried out under a pre-set strain of 0.01%, and the angular frequencies were swept from 0.006283 rad/s to 628.3 rad/s at 180 °C with a 2 mm gap. A nitrogen atmosphere was used to avoid oxidative degradation.

### 2.5. Foam Morphology Characterization

The apparent densities of unfoamed and foamed samples were calculated at room temperature. The density and expansion ratio were determined with the following two equations, respectively [22]:

(1) $$\rho =\frac{m}{\Delta m}\rho_{0},$$

(2) $$ER=\frac{\rho_{unfoamed}}{\rho_{foamed}},$$

where *ρ* and *ρ*_0_ represent the density of the sample and water at room temperature, respectively (*ρ*_0_ = 1 g/cm^3^), *m* represents the weight of the sample (g), Δ*m* represents the increased weight of a sample completely immersed in distilled water (g), and *ER* represents the expansion ratio of composite foams.

Scanning electron microscopy (SEM) of quanta 200 microscope (FEI, Hillsboro, OR, USA) was used to observe the foam morphologies. A small piece of sample was first cut from the central section of the foam. Afterwards, the samples were fractured in liquid nitrogen. Finally, the fractured surface was coated with a thin layer of gold prior to the SEM observation. ImageJ software was used to analyze the cellular information base on the SEM images. At least 200 cells were measured to obtain the average cell size of the foam. Cell density (*N*_0_) was calculated using the following Equation [30]:

(3) $$N_{0}={\left(\frac{nM^{2}}{A}\right)}^{\frac{3}{2}},$$

where *n* represents the number of cells in the micrograph, *A* represents the area, and *M* represents the magnification factor. The sample was assumed to not contain unfoamed skin in the cell density calculation.

### 2.6. Crystallization Characterization

A model Pyris Diamond device (Pekin Elmer Instruments, Waltham, MA, USA) was used to analyze the non-isothermal crystallization behavior of PP and PP/wood fiber composites. The weight of samples is 3– to 6 mg under the protection of nitrogen gas at a flow rate of about 50 mL/min. The unfoamed sample was rapidly heated from 25 to 200 °C, and then isothermally treated at 200 °C for 5 min to remove its thermal history. Subsequently, the unfoamed sample was cooled to 25 °C at constant cooling rates (5 °C/min, 10 °C/min, 20 °C/min, and 30 °C/min) and the unfoamed sample was reheated to 200 °C at the corresponding heating rates. For the foamed sample, the sample was heated from 25 to 200 °C at a heating rate of 10 °C/min. The degree of crystallinity (*χ*_c_) of PP was calculated using the following Equation [31]:

(4) $$\chi_{c}=\frac{\Delta H_{m}}{\left(1-W_{t}\right)\Delta H_{0}}\times 100\%,$$

where Δ*H*_*m*_ represents the measured melting enthalpy, Δ*H*_0_ represents the melting enthalpy of 100% crystalline PP (209.3 J/g) [31,32,33], and *W*_*t*_ represents the mass ratio of the substance (except PP).

The non-isothermal crystallization kinetics of the PP/wood fiber composites was analyzed with the following Jeziorny’s theory [34] modified Avrami equation:

(5) $$\ln[-\ln(1-X(t))]=n\ln t+\ln k_{t},$$

where *X*(*t*) represents the relative crystallinity at crystallization time *t*, *k*_*t*_ represents the crystallization kinetics constant, and *n* represents the Avrami exponent that reflects the mechanisms of both crystal nucleation and growth. ln[−ln(1−*X*(*t*))] versus ln*t* was plotted to determine *n*, and the logarithm of ln*k*_*t*_. 

Jeziorny’s theory suggests that the crystallization rate constant *k*_*t*_ should be corrected for the influence of the polymer cooling rate. Under the assumption of a constant cooling rate, the final form of the constant characterization of the kinetics of the non-isothermal crystallization was given as follows:

(6) $$\ln k_{c}=\frac{\ln k_{t}}{\Phi },$$

where *k*_c_ represents the modified crystallization rate constant considering cooling rate Φ. 

The crystallization half-time (*t*_1/2_) and the crystallization rate (*G*) were calculated via the following two equations:

(7) $$t_{1/2}={\left(\frac{\ln2}{k_{t}}\right)}^{1/n},$$

(8) $$G=\frac{1}{t_{1/2}}.$$

### 2.7. X-Ray Diffraction (XRD) Analysis

XRD patterns were obtained by a D/max2200 X-ray diffractometer (Rigaku, Tokyo, Japan) with Cu Kα as the radiation source. It was operated at 40 KV and 30 mA, and the scanning range was 10–30° (2*θ*) at a step size of 2°/min. The crystal size was calculated via the Scherrer Equation [35]:

(9) $$D=\frac{k\lambda }{\beta \cos\theta },$$

where *D* represents the crystal size (nm), *k* is a constant (0.9), λ represents the X-ray wavelength (0.154059 nm), β represents the half-width (rαd), and θ represents the diffraction angle (°).

## 3. Results and Discussion

### 3.1. Dynamic Rheological Behavior

**Figure 1:**
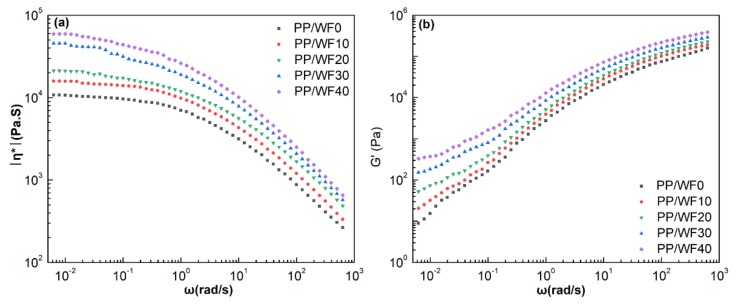
(**a**) Complex viscosity (η*) and (**b**) storage modulus (*G*’) as a function of angular frequency (ω) for (PP)/wood fiber composites at 180 °C, 0.01%, and 0.006283–628.3 rad/s.

The melt rheological behaviors of the polymer matrix played a central role in the foaming process, as it tailored both cell growth and cell stabilization [20]. Therefore, the viscoelasticity of an ideal foaming system should be controlled within a certain range. In the present work, the effects of the wood fiber on the melt rheology and the processability of PP were examined via dynamic viscosity measurements. As shown in Figure 1, the complex viscosity (η*) of the composites decreased with the angular frequency (ω). All samples displayed typical shear thinning behaviors in the measured frequency range. In the low frequency region, the complex viscosity of PP/wood fiber composites improved with increasing wood fiber content [16,19,24]. Virgin PP showed the lowest viscosity compared to other samples in the whole frequency range. The wood fiber helped to increase polymer rigidity, the collision probability of the wood fibers, and the interaction between PP molecular chains and wood fibers. In addition, wood fibers were easily aggregated in the polymer matrix as a result of inter-fiber hydrogen bonding and physical entanglement. Both perturb the normal flow of the PP matrix and hinder the mobility of the chain segments in the direction of flow. This improves the viscosity of the PP/wood fiber composites, which in turn results in a decreased cell growth rate and high cell density [35].

Figure 1b depicts the storage modulus (*G*’) of the PP/wood fiber composites as a function of angular frequency. As shown in Figure 1b, the storage modulus improved with increasing wood fiber loadings. For polymer systems filled with wood fibers, the increasing fiber concentration led to the enhanced discontinuity of the matrix, which may result in the squeezing out of the polymer to the surface, creating continuity on the surface. This continuity facilitates elastic energy recovery and increases the melt elasticity [36]. Moreover, further increasing the wood fiber loadings to 40 wt.%, the composites were less frequency-dependent at low frequencies, indicating the possible formation of a network microstructure in PP/wood fiber composites, which restrained the relaxation of the PP molecular chains. This phenomenon illustrated that the viscoelastic response changed from a pseudo plastic-like to a pseudo solid-like behavior [22,23].

### 3.2. Non-Isothermal Crystallization Behavior

**Figure 2:**
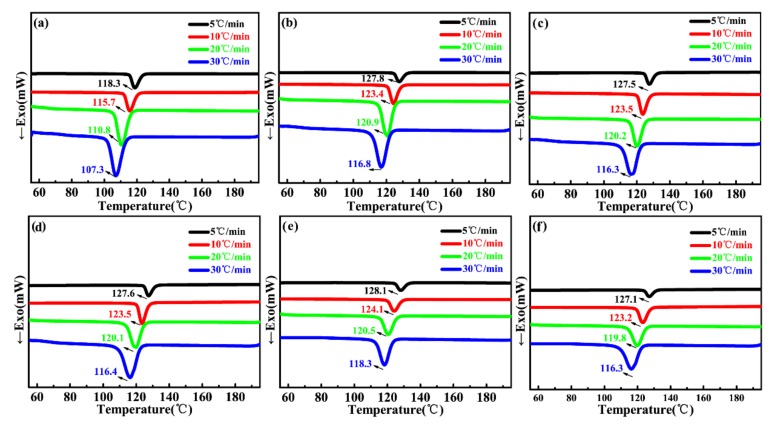
The non-isothermal crystallization of unfoamed neat PP and its composites at various cooling rates: (**a**) Neat PP, (**b**) PP/WF0, (**c**) PP/WF10, (**d**) PP/WF20, (**e**) PP/WF30, and (**f**) PP/WF40.

The non-isothermal crystallization exotherms of neat PP and PP/wood fiber composites recorded at various cooling rates are shown in Figure 2a–f. As shown in Figure 2, with increasing cooling rate, the onset crystallization temperature, and the crystallization temperature shifted toward lower temperatures. This was because the movement of PP molecular chains was restrained at higher cooling rates and crystallization took place at lower temperatures. However, sufficient time contributes to the nucleation at lower cooling rates, resulting in crystallization at higher temperatures [37,38]. Compared to neat PP, the incorporation of MAPP, PE wax, and Talc could globally increase the crystallization temperature of PP by 10 °C. The shifts to higher crystallization temperatures indicate the acceleration of the overall PP crystallization. However, there was no obvious change in the crystallization temperature at various cooling rates with increasing wood fiber loadings. In a previous study, a suitable concentration of wood fibers acted as heterogeneous nucleation sites by providing a surface for the crystal nucleation like Talc [39,40]. This led to an increase in the crystallization temperature [39]. However, the high wood fiber loadings induced a higher resistance to the mobility of the PP molecular chains under the presence of MAPP, which could decrease the movement of the PP segment, resulting in a lower crystallization temperature. Overall, both contradicting effects maybe have resulted in a non-obvious change in crystallization temperature.

**Figure 3:**
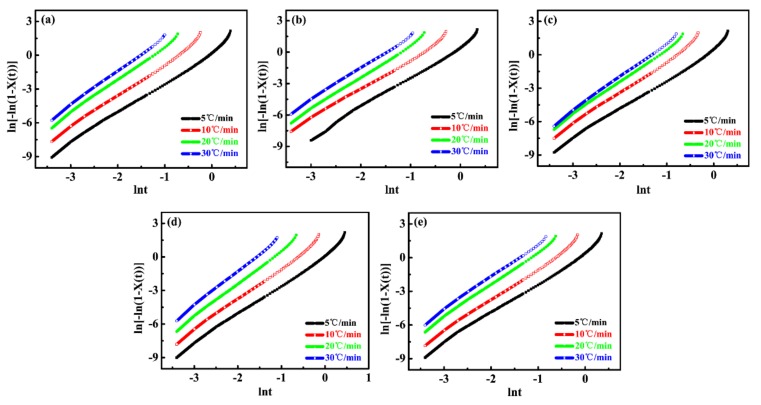
Avrami double-log plots for the non-isothermal crystallization of the unfoamed PP/wood fiber composites at various cooling rates: (**a**) PP/WF0, (**b**) PP/WF10, (**c**) PP/WF20, (**d**) PP/WF30, and (**e**) PP/WF40.

Figure 3a–e displays the Avrami double-log plot for PP/wood fiber composites at various cooling rates. The half-time of crystallization and other crystallization parameters obtained from the Avrami plots and Jeziorny’s methods are listed in Table 1. As illustrated in Figure 3, the fitted lines were approximately parallel at various cooling rates, indicating that the crystal nucleation mechanism and growth morphology were similar for various cooling rates [34]. As calculated in Table 1, the Avrami exponent *n* was close to 3 for all samples. Moreover, the nucleation was heterogeneous induced by both wood fiber and Talc. Therefore, the crystals mainly grew as a 2D crystal plate and a 3D spherule during crystallization [34,41], which illustrated that wood fibers did not change the crystal growth. In addition, when the cooling rate was below 10 °C/min, the increase in cooling rate enhanced the modified crystallization rate constant *k*_c_. However, under the same cooling rate, the addition of wood fibers could reduce the crystallization rate, which was in agreement with the calculated results according to Equations (7) and (8). The wood fibers could act as heterogeneous nucleation sites and improve crystal nucleation. However, in high wood fiber loadings, the complex viscosity of the composites significantly increased (Figure 1). Therefore, the mobility of the PP molecular chains was greatly impeded during crystallization, causing a decreased crystal growth rate. In this case, the global crystallization rate was dominated by the crystal growth. As a result, the increasing wood fiber loadings slowed the crystallization kinetics of the PP matrix.

### 3.3. Apparent Density and Expansion Ratio of Composite Foams

**Figure 4:**
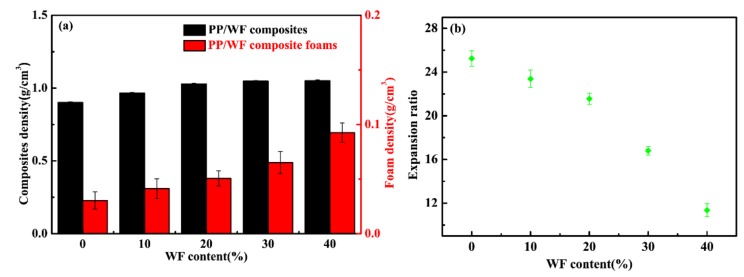
(**a**) Apparent density of the PP/wood fiber composites and the corresponding composite foams and (**b**) expansion ratio of the composite foams.

Figure 4a shows the apparent density of the original unfoamed composites and the microcellular foamed PP/wood fiber composites as a function of wood fiber content. The apparent density of the unfoamed PP/wood fiber composites increased with increasing wood fiber loadings. The density (1.30–1.50 g/cm^3^) of the solid wood substance was larger than that of PP (0.85–0.95 g/cm^3^), although the bulk density of wood fibers was small. After compression molding, the wood cell walls are compressed nearly to the specific solid wood and the voids between and within the wood-fiber structure are completely filled with resin. Compared to unfoamed composites, the apparent density of the composite foams was noticeably reduced. However, the reduced amplitude showed a decreased trend with increasing wood fiber content. When the wood fiber content increased further, the apparent density of the PP/wood fiber composite foams increased, due to their weakened foamability.

As shown in Figure 4b, the expansion ratio decreased with increasing wood fiber loadings, indicating that incorporation of wood fiber affected the foamability of PP. On the one hand, wood fibers increased the complex viscosity of the composites (Figure 1), which indicated an increased resistance for cell growth [40]. On the other hand, the higher wood fiber content indicated less PP matrix available for gas solution. As CO_2_ could only dissolve in the amorphous portion of the PP matrix during the saturation stage, CO_2_ could only diffuse into the amorphous portion of the PP matrix to induce the cell growth during the rapid-decompression foaming. As a result, the solubility of the scCO_2_ decreased and gas escaping occurred. Most of the bubbles that were developed during the cell growth were not preserved, which in turn decreased the expansion ratio of the PP/wood fiber composite foams.

### 3.4. Cell Morphology

**Figure 5:**
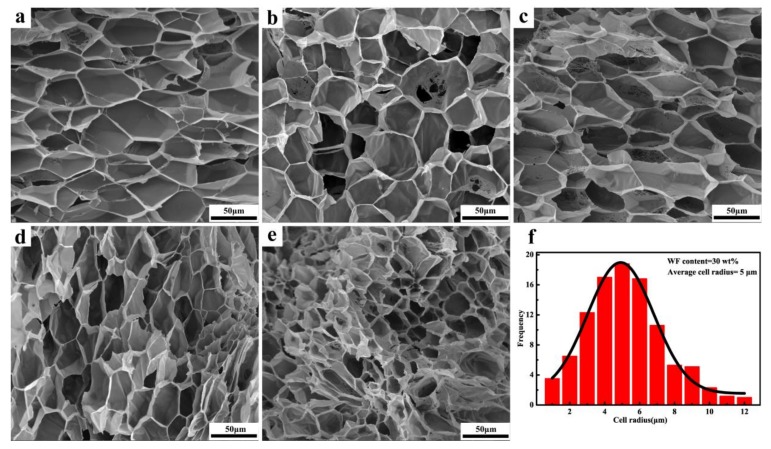
Scanning electron microscopy (SEM) micrographs of the fracture surface of PP/wood fiber composite foams with various wood fiber contents: (**a**) PP/WF0, (**b**) PP/WF10, (**c**) PP/WF20, (**d**) PP/WF30, (**e**) PP/WF40, and (**f**) cell size distribution of composite foams with 30 wt.% wood fibers.

The cell morphology of the PP/wood fiber composite foams is illustrated in Figure 5. The cells of composite foams had cellular structures similar to those of neat PP foams. As wood fiber contents increased, the average cell size decreased compared to the neat PP foam. The reduced cell size may be attributed to the poor interfacial adhesion between the PP matrix and wood fibers, which provided channels for easy and quick gas escape to the environment during cell growth. In addition, the aforementioned melt rheology behaviors indicated that incorporating wood fiber could improve the melt viscosity of the PP matrix (Figure 1a), which provides higher resistance to the cell growth in the polymer matrix. However, the cell density was improved in the presence of wood fibers. Previous studies reported that wood fibers could provide heterogeneous nucleation sites [20,21]. Cell nucleation tended to occur at the interface between the PP matrix and wood fibers during the foaming process, due to the low nucleation energy barrier [40]. Therefore, higher wood fiber loadings led to more heterogeneous nucleation sites, which resulted in a markedly higher cell density. As shown in Figure 5f, when the wood fiber loadings were 30 wt.%, the cell size distribution was narrow, the average cell radius decreased to 5 μm, and the cell density increased to 10^9^ cells/cm^3^ calculated from Figure 5d. These results indicate that incorporating wood fibers into the PP matrix produced the expected effect. The uniform cell size distribution was attributed to the decreased energy barrier induced by the wood fiber. However, when the wood fiber content was further increased to 40 wt.%, the cell structure began to deteriorate, displaying cell coalescence, cell rupture, and irregular cell shape, which indicated that 30 wt.% was likely the threshold of wood fiber loadings for the well-developed PP/wood fiber foaming system [20,42]. Excessively high wood fiber loadings resulted in the agglomeration phenomenon, which in turn led to a non-uniform cell size distribution. Furthermore, excessive wood fibers resulted in poor interface compatibility between wood fibers and the PP matrix, resulting in the formation of the interfacial channel and poor expandability. In addition, the wood fibers destroyed the continuous phase structure of the polymer matrix, leading to cell coalescence and cell rupture [21].

**Figure 6:**
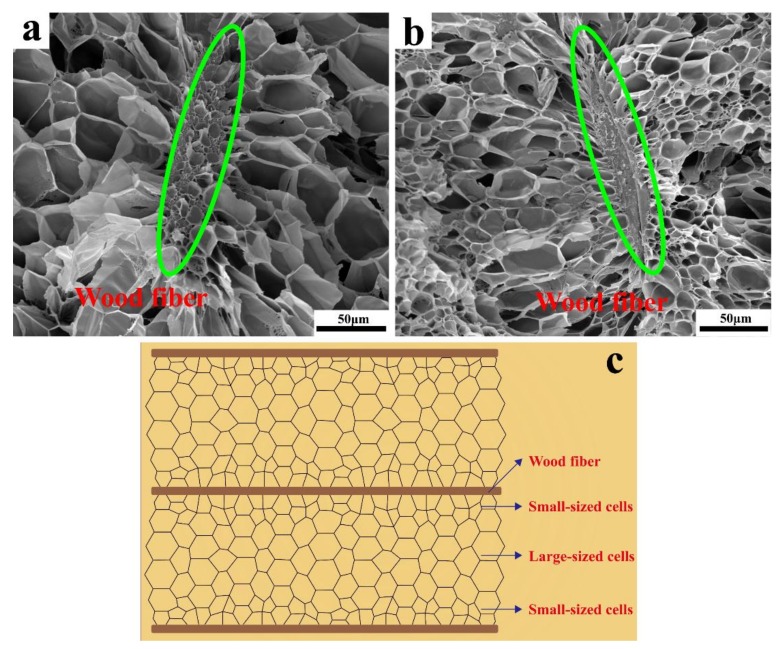
Cell morphology around the wood fibers: (**a**) 20 wt.% wood fibers, (**b**) 30 wt.% wood fibers, and (**c**) schematic diagram of “small-sized cells to large-sized cells” gradient cell distribution characteristics surrounding the wood fibers.

To gain an improved understanding of the cell nucleation mechanism around the boundary of the PP matrix and the wood fibers, the cell morphology around the wood fibers was investigated. As illustrated in Figure 6 (composite foams with 20 and 30 wt.% wood fibers), cells grew along the wood fiber surface. These cells had smaller cell size near the wood fibers, indicating that the wood fiber had a significant effect on the cell nucleation due to its large surface area. During the cell nucleation stage, the wood fibers acted as heterogeneous nucleation sites, which increased the cell density of the composite foams. The cell size was larger in the region that was further from the wood fiber surface, which could be attributed to the lower viscosity distribution of the PP matrix and the corresponding low resistance for cell growth [20,21]. Figure 6c depicts “the small-sized cells to large-sized cells” diversified cell size distribution characteristics around the wood fibers. Previous experiments and simulations showed that three dynamic components of polymer chains in filled polymer composites could be observed. These components were: Tightly bound chains, loosely bound chains, and mobile chains [43,44]. Therefore, the mobility of the PP molecular chains presented a gradient distribution, which in turn led to the gradient viscoelasticity distribution around the wood fibers [45].

### 3.5. Crystallinity and Melting Behaviors of Composite Foams

**Figure 7:**
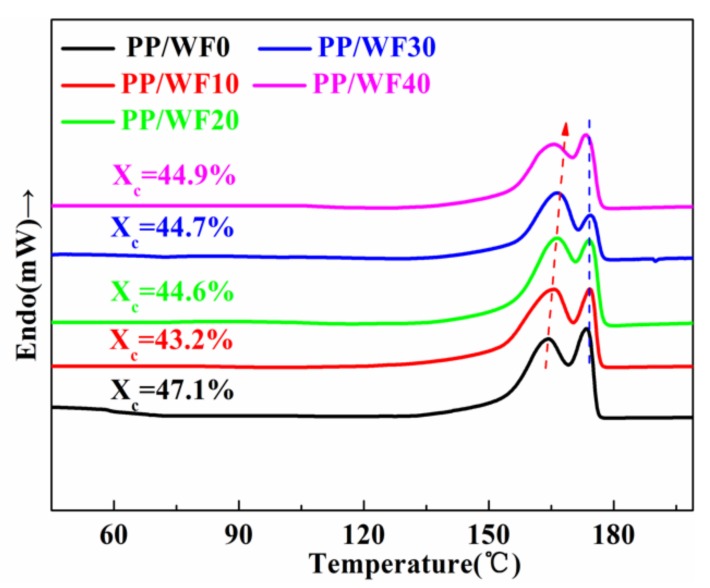
DSC curves of PP/wood fiber composite foams with various wood fiber loadings.

Figure 7 shows the melting behavior of PP/wood fiber composite foams at various wood fiber loadings. All samples had double melting peaks. This phenomenon was common for semi-crystallization polymers, which resulted from the melt-reorganization of less ordered crystals [23]. Crystallization is a complex process, and the crystallization and the crystal growth differed between perfect crystals and imperfect crystals. During the crystal nucleation stage, perfect crystals were dependent on thermal motion, whereas imperfect crystals were dependent on tensile stress [46]. Therefore, the phenomenon of the double melting peaks was dependent on the tensile stress derived from the cell growth and the dissolved scCO_2_. The imperfect crystals have poor thermal stability. Therefore, the low temperature peak corresponded to the melting of imperfect crystals, and the higher temperature peak corresponded to the melting of perfect crystals. In addition, as the wood fiber contents increased, the low melting temperature peak moved to a higher temperature. The crystallinity initially decreased and then increased (Table 2) and the double melting peaks phenomenon gradually disappeared, which could be attributed to the poor foamability (Figure 4b) induced deficient crystallization driving force in high wood fiber loadings.

### 3.6. Crystal Structure of the Composite Foams

**Figure 8:**
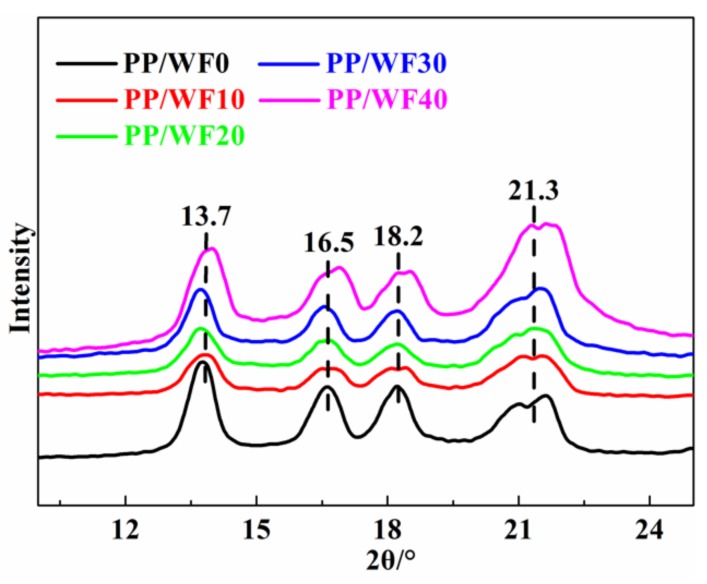
X-ray diffraction patterns of the PP/wood fiber composite foams with various wood fiber loadings.

The DSC curves show that the PP/wood fiber composite foams had two melting peaks, whereas the XRD curves (Figure 8) show that the foamed composites exhibited diffraction peaks at around 2θ = 13.7°, 16.5°, 18.2°, and 21.3°. These peaks were in agreement with the α-crystal of the PP matrix [39] and could be attributed to the reflections of (110), (040), (130), and (111) planes [47], respectively, indicating that there was no new crystalline formation. However, the XRD pattern showed that the wood fibers changed both peak broadness and intensity. The larger peak broadness and the higher intensity corresponded to the looser packing of the crystalline structure and the higher crystallinity, respectively. Table 3 shows the crystal size of the foamed composites with various wood fiber loadings. Both the crystal size of each crystal plain and the average crystal size decreased when the wood fiber loadings increased, illustrating that the wood fiber could increase the heterogeneous nucleation sites, leading to the reduced crystal size of the composite foams [24]. Moreover, the wood fibers hindered the mobility of the PP molecular chains. Therefore, the crystal growth was restrained. Then, the crystal nucleation domain phenomenon was present during the crystallization behavior, thus resulting in small-sized crystals. As a result, the broader peak and a loosely packed crystalline structure were obtained as wood fiber loadings increased.

## 4. Conclusions

Microcellular PP/wood fiber composite foams, with desirable cell structure, were prepared via batch foaming. The wood-fiber-induced crystallization, rheology, and the microcellular foaming behaviors of the PP/wood fiber composites were investigated. The incorporation of wood fibers improved the complex viscosity and the storage modulus of the PP matrix. However, the viscoelastic response of the composites changed from a pseudo plastic-like to a pseudo solid-like behavior for high wood fiber loadings. Jeziory’s theory was successfully used to predict the crystallization kinetics constant after taking the cooling rate into account. Wood fibers did not change the crystal growth, whereas the addition of wood fiber reduced the overall crystallization rate to a certain extent. Compared to neat PP foams, the PP/wood fiber composite foams had a smaller average cell size and a higher cell density despite their lower expansion ratio. Furthermore, cell nucleation occurred at the interface between the wood fibers and the PP matrix. A “large-sized cells to small-sized cells” diversified cell distribution was observed around the wood fibers, which could be attributed to the fact that the viscoelasticity presented a gradient distribution around the wood fibers. When the wood fiber loadings reached 30 wt.%, desirable microcellular PP/wood fiber composite foams were prepared: The apparent density was 0.0652 g/cm^3^, the cell radius was about 5 μm, and the cell density reached 10^9^ cells/cm^3^. Further increasing the wood fiber loadings disrupted the cell structure, including cell coalescence and cell collapse. Our study made it possible to develop polymer/wood fiber composite foams with high expansion ratio and desirable cell structure in the high wood fiber loadings, which will in turn provide direction for the continuous production of PP/wood fiber composite foams.

## Figures and Tables

**Table 1:** Crystallization half-time, Jeziorny’s theory modified crystallization rate constant, and Avrami parameter of unfoamed PP/wood fiber composites at various cooling rates.

Samples	Φ (°C/min)	*t*_1/2_ (min)	*G* (min^−1^)	*n*	ln*k*_t_	ln*k*_c_	*k*_c_
PP/WF0	5	0.66	1.52	2.76	0.75	0.15	1.16
	10	0.36	2.78	2.89	2.60	0.26	1.30
	20	0.14	7.14	2.95	5.40	0.27	1.31
	30	0.07	14.29	3.02	7.50	0.25	1.28
PP/WF10	5	0.74	1.35	2.93	0.50	0.10	1.11
	10	0.38	2.63	2.89	2.40	0.24	1.27
	20	0.21	4.76	3.09	4.40	0.22	1.25
	30	0.11	9.09	3.02	6.30	0.21	1.23
PP/WF20	5	0.76	1.32	2.76	0.40	0.08	1.08
	10	0.44	2.27	2.91	2.00	0.20	1.22
	20	0.26	3.85	2.95	3.60	0.18	1.20
	30	0.13	7.69	3.02	5.70	0.19	1.21
PP/WF30	5	0.78	1.28	2.72	0.30	0.06	1.06
	10	0.50	2.00	2.85	1.60	0.16	1.17
	20	0.30	3.33	2.99	3.20	0.16	1.17
	30	0.17	5.88	3.09	5.20	0.14	1.15
PP/WF40	5	0.81	1.23	2.75	0.20	0.04	1.04
	10	0.52	1.92	2.87	1.50	0.15	1.16
	20	0.36	2.78	2.91	2.60	0.13	1.14
	30	0.25	4.00	2.90	3.60	0.12	1.13

**Φ**: cooling rate, ***t*_1/2_**: crystallization half-time, ***G***: crystallization rate, ***n***: Avrami exponent, **ln*k*_c_**: logarithm of the modified kinetics constant, ***k*_c_**: modified crystallization kinetic constant.

**Table 2:** Melting parameters of the foamed PP/wood fiber composites with various wood fiber loadings.

Samples	*T*_L_ (°C)	*T*_H_ (°C)	*X*_c_ (%)
PP/WF0	164.3	173.5	47.1
PP/WF10	165.8	174.2	43.2
PP/WF20	166.6	174.3	44.6
PP/WF30	166.8	174.8	44.7
PP/WF40	166.2	173.4	44.9

*T*_L_: Low melting temperature peak; *T*_H_: High melting temperature peak; *X*_c_: Degree of crystallinity.

**Table 3:** Crystal size of PP/wood fiber composite foams with various wood fiber loadings.

Samples	Crystal Grain Size/nm				Average Size
	[110]	[040]	[130]	[111]	
PPWF0	12.38	10.28	9.43	5.82	9.48
PP/WF10	10.52	10.16	8.48	6.81	8.99
PP/WF20	10.42	10.25	9.45	6.06	9.05
PP/WF30	11.13	7.42	9.02	6.40	8.49
PP/WF40	10.50	7.43	8.46	5.09	7.87

The crystal grain size was calculated with the Scherrer equation and the average size indicates the mean value of the four crystal planes.
